# Long-Term Survival after Linac-Based Stereotactic Radiosurgery and Radiotherapy with a Micro-Multileaf Collimator for Brain Metastasis

**DOI:** 10.3390/curroncol29090477

**Published:** 2022-08-24

**Authors:** Ryosuke Matsuda, Masatoshi Hasegawa, Tetsuro Tamamoto, Nobuyoshi Inooka, Mei Nikimoto, Tomoko Ochi, Toshiteru Miyasaka, Shigeto Hontsu, Kaori Yamaki, Sachiko Miura, Takayuki Morimoto, Takaaki Mitsui, Takanori Furuta, Shohei Yokoyama, Masashi Kotsugi, Shuichi Yamada, Ichiro Nakagawa, Young-Soo Park, Hiroyuki Nakase

**Affiliations:** 1 Department of Neurosurgery, Nara Medical University, Kashihara 634-8521, Japan; 2 Department of Radiation Oncology, Nara Medical University, Kashihara 634-8521, Japan; 3 Department of Medical Informatics, Nara Medical University Hospital, Kashihara 634-8522, Japan; 4 Department of Radiology, Nara Medical University Hospital, Kashihara 634-8522, Japan; 5 Department of Respiratory Medicine, Nara Medical University Hospital, Kashihara 634-8522, Japan

**Keywords:** linac with a micro-multileaf collimator, brain metastasis, long-term survivors, stereotactic radiosurgery, stereotactic radiotherapy

## Abstract

Background: this study aimed to evaluate the prognostic factors associated with long-term survival after linear accelerator (linac)-based stereotactic radiosurgery (SRS) and fractionated stereotactic radiotherapy (fSRT) with a micro-multileaf collimator for brain metastasis (BM). Methods: This single-center retrospective study included 226 consecutive patients with BM who were treated with linac-based SRS or fSRT with a micro-multileaf collimator between January 2011 and December 2018. Long-term survival (LTS) was defined as survival for more than 2 years after SRS/fSRT. Results: The tumors originated from the lung (*n* = 189, 83.6%), breast (*n* = 11, 4.9%), colon (*n* = 9, 4.0%), stomach (*n* = 4, 1.8%), kidney (*n* = 3, 1.3%), esophagus (*n* = 3, 1.3%), and other regions (*n* = 7, 3.1%). The median pretreatment Karnofsky performance scale (KPS) score was 90 (range: 40–100). The median follow-up time was 13 (range: 0–120) months. Out of the 226 patients, 72 (31.8%) were categorized in the LTS group. The median survival time was 43 months and 13 months in the LTS group and in the entire cohort, respectively. The 3-year, 4-year, and 5-year survival rate in the LTS group was 59.1%, 49.6%, and 40.7%, respectively. Multivariate regression logistic analysis showed that female sex, a pre-treatment KPS score ≥ 80, and the absence of extracranial metastasis were associated with long-term survival. Conclusions: female sex, a favorable pre-treatment KPS score, and the absence of extracranial metastasis were associated with long-term survival in the current cohort of patients with BM.

## 1. Introduction

Brain metastasis (BM) is the most common intracranial brain neoplasm, found in 8% to 35% of cancer patients [1]. BMs may be discovered at the same time as the primary tumor is diagnosed, or they may be newly discovered during or after treatment. As a result of advances in chemotherapy and radiotherapy against cancers, there are several treatment options, including whole-brain radiotherapy, surgical resection, and stereotactic radiosurgery (SRS) or fractionated stereotactic radiotherapy (fSRT). Among these modalities, SRS and fSRT for brain metastasis are associated with good tumor control, low toxicity, and few complications [2]. In our previous studies, SRS/fSRT with a frameless fixation system was used to treat brainstem metastasis, BM in the primary motor cortex, and large brain metastasis with an unsuitable surgical resection, and showed good tumor control with the possibility of reducing radiation necrosis [3,4,5]. The long-term survival outcomes after this procedure may be related to the pre-radiosurgical characteristics of the patients. However, to the best of our knowledge, there are very few studies on the factors associated with long-term survival after SRS/fSRT for BM [6,7,8,9,10]. Therefore, this retrospective study aimed to identify the clinical and radiographic factors associated with long-term survival after linear accelerator (linac)-based SRS and fSRT for BM.

## 2. Materials and Methods

### 2.1. Patient Characteristics

This retrospective study was approved by the ethical committee of our hospital (Approval No. 2924). Clinical data were retrospectively collected from 243 consecutive patients who were treated with SRS and fSRT at our hospital between January 2011 and December 2018. The most suitable modality for each patient was determined by a multidisciplinary team comprising neurosurgeons, neuro-oncologists, neuroradiologists, and radiation oncologists. In total, 17 patients were lost before follow-up during this period and were excluded from analysis. Finally, 226 patients were included, and Table 1 shows the characteristics of all these patients. The cohort comprised 140 men and 86 women, with a median age of 68.6 ± 10.3 years. The primary tumor originated from the lung (*n* = 189, 83.6%), breast (*n* = 11, 4.9%), colon (*n* = 9, 4.0%), stomach (*n* = 4, 1.8%), kidney (*n* = 3, 1.3%), esophagus (*n* = 3, 1.3%), and other regions (*n* = 7, 3.1%).

### 2.2. SRS and fSRT

The SRS and fSRT plans were based on CT scans with a slice thickness of 1 mm. All patients were immobilized with a thermoplastic mask (BRAINLAB AG, Munich, Germany). The gross tumor volume (GTV) for each lesion was delineated on MRI scans with a slice thickness of 1 mm. The planning target volume (PTV) was defined as the volume 1–2 mm around the GTV. Treatment was provided within 1 week after treatment planning based on the CT scan. Treatment planning was performed using BrainSCAN or iPlan RT (BRAINLAB AG). The irradiation dose was prescribed so as to ensure a dose coverage of 90% for the PTV. Dose calculations were performed using a pencil beam algorithm. SRS and fSRT were performed using linacs with a micro multi-leaf collimator: Novalis*^®^* (BRAINLAB AG) with a collimator width of 3 mm or TrueBeam STx (Varian Medical Systems, Palo Alto, CA, USA) with a collimator width of 2.5 mm. Patients were treated with Novalis until November 2017, and patients have been treated with TrueBeam STx since November 2017. Every patient was treated with X-rays of 6 MV beam energy. Patient positioning and verification were performed using BrainLab ExacTrac*^®^* (BRAINLAB AG). This device comprises two infrared cameras and two dual diagnostic kV X-ray tubes, which can be moved automatically into the required position to minimize setup errors [11,12]. Basically, patients with neurological symptoms, with brain metastases larger than 20 mm in size, and with brain metastases in the eloquent area including basal ganglia and primary motor cortex [5] underwent fSRT in the doses of 35 Gy in 5 fractions. Patients with brainstem metastasis were treated with in the doses 24–40 Gy in 7–13 fractions [3]. Asymptomatic patients with brain metastases smaller than 20 mm were treated with SRS in the doses of 21–22.5 Gy in a single fraction, and the decision was based on tumor size, location, surrounding edema, and other factors. All patients were treated using Novalis*^®^* or TrueBeam STx via non-coplanar multi-beams, non-coplanar multi-arcs, or both. For both SRS or fSRT, conformal beams, dynamic conformal arcs, intensity-modulated radiotherapy (IMRT), or hybrid arcs were used. This is a novel treatment technique that blends aperture-enhanced optimized arcs with discrete IMRT elements, thereby allowing arc selection with a set of static IMRT beams [13].

### 2.3. Clinical and Radiological Follow-Up

Follow-up contrast-enhanced MRI was performed every 3 months after the end of SRS/fSRT if possible. Tumor volumes were evaluated before and after SRS/fSRT using BrainSCAN or iPlan RT. Once disease progression was detected, MRI was performed at intervals of 1–2 months. The decision for additional treatment for disease progression was based on evidence of clinical deterioration and associated imaging progression judged by the inter-disciplinary team. Local failure was defined as either a pathologically proven recurrence within the SRS/fSRT treatment field. Imaging progression that triggered additional treatment (surgery for symptomatic radiation necrosis, SRS/fSRT, or medication including steroid and bevacizumab) were counted as local failure without pathological confirmation. Distant failure was defined as intracranial failure at a site not previously treated with SRS/fSRT. Long-term survival (LTS) was defined as survival for more than 2 years after SRS/fSRT, and short-term survival (STS) was defined as death within 2 years after SRS/fSRT.

### 2.4. Statistics

The median survival time was calculated using the Kaplan–Meier method. The log-rank test was used for univariate analyses. We analyzed the prognostic factors age, sex, tumor volume, extracranial metastasis, control of the primary cancer, pretreatment Karnofsky performance scale (KPS) score (≥80 vs. <80), treatment method (SRS vs. SRT), and tumor volume. Univariate and multivariate regression logistic analyses were used to identify factors associated with survival. All analyses were performed with the EZR software (Saitama Medical Center, Jichi Medical University, Saitama, Japan) [14], and a *p* value of <0.05 was considered to indicate statistical significance.

## 3. Results

### 3.1. Patient Characteristics in Both Groups

In total, 226 patients underwent SRS and fSRT for 733 BMs during the study period. Based on survival, 72 patients (31.8%) were assigned to the LTS group, and 154 patients (68.1%), to the STS group. In the LTS group, 37 and 35 patients were male and female, respectively, with a median age of 67.6 ± 9.6 years. In the STS group, 103 and 51 patients were male and female, respectively, with a median age of 69.0 ± 10.5 years. With regard to the KPS score, 66.2% of the patients in the STS group and 86.1% in the LTS group had a KPS score ≥ 80. In the STS group, 78.6% of BMs originated in the lungs, and in the LTS group, 94.4% originated in the lungs. At the initial SRS or fSRT, 55.2% patients in the STS group had a single BM and 63.4% in the LTS group had multiple BMs. Further, 33.8% patients in the STS group and 56.9% in the LTS had well-controlled primary cancer, and 51.3% patients in the STS group and 27.8% patients in the LTS group had extracranial metastasis. Based on recursive partition analysis (RPA), 5, 105, and 44 patients in the STS group were assigned to classes I, II, and III, respectively, while 8, 55, and 9 patients in the LTS group were assigned to classes I, II, and III, respectively. Significant differences were found between the LTS and STS group with regard to sex (*p* = 0.028), pretreatment KPS score (*p* = 0.002), tumor origin (*p* = 0.002), mutation status of lung cancer (*p* = 0.048), control of primary cancer (*p* = 0.001), extracranial metastasis (*p* < 0.001), and RPA classification (*p* = 0.001) (Table 2).

### 3.2. Treatment Outcomes

**Figure 1:**
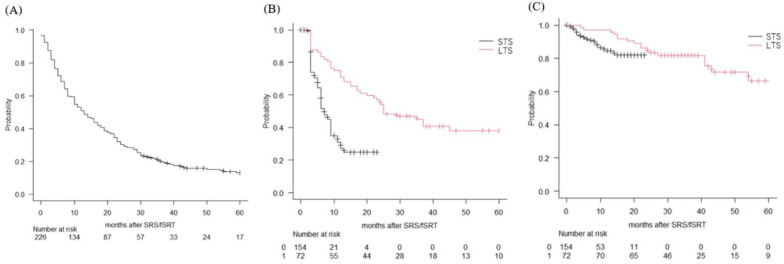
Treatment outcomes. Overall survival of the entire cohort of patients with brain metastases (**A**), distant failure of the STS (black line) and LTS (red line) groups (**B**), local failure of the STS (black line) and LTS (red line) groups (**C**) estimated using the Kaplan–Meier method.

During the study period, all the patients in the STS group died within 2 years after the initial SRS or SRT (as per the cut-off for this group). Of the 154 patients in the STS group, 139 patients died due to worsening of the primary cancer and 16 patients died due to the BM. At the last follow-up, of the 72 patients in the LTS group, 43 patients died due to worsening of the primary cancer and 1 died due to the BM. The median survival time of the entire cohort and those with BMs originating from the lung was 13 and 15 months, respectively. The median survival time in the LTS group was 43 months (95% confidence interval = 36–73 months). The 3-year, 4-year, and 5-year survival rate in the LTS group was 59.1%, 49.6%, and 40.7%, respectively. Freedom from distant failure at 1 year was 27%, and 70.8% for the STS and the LTS, respectively. Freedom from local failure at 1 year was 84.6%, and 97.2% for the STS and the LTS, respectively. In the LTS group, freedom from distant and local failure at 2 years were 54.2% and 83.3%, respectively (Figure 1).

During the study period, 7 (4.5%) of 154 patients in the STS group underwent salvage surgical resection. In total, 5 patients underwent surgical resection within 6 months of SRS or fSRT, 1 patient at 6 to 12 months, and 1 patient after 1 year. Among them, 6 patients had tumor recurrence, and 1 patient had radiation necrosis. On the other hand, in the LTS group, 13 (18%) of the 72 patients underwent salvage surgical resection. In total, 2 patients underwent surgical resection within 12 months of SRS or fSRT, 4 patient at 12 to 24 months, and 7 patients after 2 years. The histological findings for the LTS group indicated recurrence in 7 cases, radiation necrosis in 5 cases, and cyst formation in 1 case. This number of patients who underwent salvage surgical resection was significantly higher (*p* = 0.002) than that in the STS group.

### 3.3. Factors Associated with Survival

Logistic regression analysis was used to identify the factors associated with long-term survival. Female sex (odds ratio = 1.94, *p* = 0.034), pre-treatment KPS score ≥ 80 (odds ratio = 2.54, *p* = 0.021), and absence of extracranial metastasis (odds ratio = 2.35, *p* = 0.019) (Table 3) were associated with LTS.

## 4. Discussion

### 4.1. Long-Term Survival after SRT/fSRT for BM

The present study examines the factors associated with long-term survival in patients with BM who have undergone linac-based SRT and fSRT with micro-multileaf collimators. In the study cohort, the median survival time was 13 months in all the patients, and 72 patients (31.9% of the entire cohort) survived for more than 2 years after SRS and fSRT and were included in the LTS group. In the LTS group, the median survival time after initial SRS/fSRT was 43 months, and the 3-year, 4-year, and 5-year survival rate was 59.1%, 49.6%, and 40.7%, respectively. The prognostic factors associated with LTS were female sex, good pre-treatment KPS score (≥80), and absence of extracranial metastasis before SRS and fSRT. In a previous report, Cacho Díaz et al. demonstrated that the prognostic factors associated with long-term survival were female sex, single metastasis, brain metastasis at the initial diagnosis, and location of the metastasis in the occipital lobe, but their study included all treatment modalities, such as whole brain radiotherapy, surgical resection, SRS, and systemic/intrathecal chemotherapy. In another such study, Dasgupta et al. reported that patients who were younger than 60 years and had a single BM survived for more than 3 years. The patients in their study received surgical, targeted, immune, or hormonal therapy. However, they had summarized the results of previous researchers, who had adopted widely varying definitions of long-term survival that ranged from ≥2 years to ≥10 years. Therefore, it is difficult to definitively determine the prognostic factors for LTS because of these differences in treatment modalities and definitions of long-term survival across the published studies [15].

Table 4 summarizes the published data on long-term survival in patients with BM treated with SRS and fSRT [6,7,8,9,10]. Although the definitions of long-term survival and the aims of the studies differed, the trend across the reports could be observed. The overall survival time ranged from 25.2 to 68 months, and the overall survival rate at 3 years and 5 years was 33.4–73.7% and 16.6–41.7%, respectively. Based on the data, it seems that survival beyond 1–4 years after SRS and fSRT resulted in even longer survival. Sato et al. scrutinized factors affecting long-term survival after stereotactic radiotherapy for metastatic brain tumors and further developed a scoring system based on these factors. The five factors they proposed were number of metastases, gender, KPS, primary tumor, control of primary tumor, and extracranial metastases. Patients were divided into four groups according to the total score, which was reflected in OS [16].

In this study, a significantly higher number of patients in the LTS group compared to the STS group required salvage surgery. Other studies have also reported recurrence, radiation necrosis, and cyst formation during long-term follow-up after SRS and fSRT [17,18,19,20,21,22]. Table 4 shows that the rate of salvage surgery after SRS and fSRT in the LTS groups ranged from 5.1% to 18.0% in the reported studies. Further, in accordance with the present findings, the long-term follow-up results show that there were relatively more cases of relapse in the LTS patients. Based on these findings, in patients who have survived for more than 1–4 years after SRS and fSRT, it is important to pay close attention to the possibility of recurrence and need for salvage surgery and continue with detailed imaging follow-up.

### 4.2. Limitations

The main limitations of the present study are its retrospective design and the lack of a standard definition of long-term survival across published studies.

## 5. Conclusions

Female sex, better pre-treatment KPS score, and absence of extracranial metastasis were associated with long-term survival. Further, a higher number of patients in the LTS group than in the STS group required re-operations. This data suggests ongoing close follow up with regular MRI scans is justified due to local and distant failures in LTS.

## Figures and Tables

**Table 1:** Characteristics of patients with brain metastases and survival.

Characteristic	Number	Median Survival Time (Months)	Log-Rank Test
Sex			
	Male 140	10	0.045
	Female 86	17.5	
Age(years)			
	Median (range) 68.6 ± 10.3	age ≥ 70 12.0	0.126
		age < 70 13.5	
Pretreatment KPS			
	Median (range) 90 (40–100)	KPS ≥ 80 17.0	<0.0001
		<80 5.5	
Tumor origin			
lung/breast/colon/stomach/kidney/esophagus/others	189/11/9/4/3/3/7	lung 15.0	<0.0001
		non- long 5.0	
Tumor number			
	single 131	single 16.0	0.173
	multiple 95	multiple 10.0	
Control of primary tumor			
	yes 93	yes 21.0	<0.0001
	no 133	no 8.0	
Extracranial metastasis			
	yes 99	yes 7.0	<0.0001
	no 127	no 19.0	
RPA class			
	I 13	I 29.0	<0.0001
	II 160	II 16.0	
	III 53	III 5.0	

**Table 2:** Characteristics of the STS and LTS groups.

Characteristic	Survival <24 Months	Survival ≥24 Months	*p*-Value
	*n* = 154	*n* = 72	
Sex			
Female	51	35	0.028
Male	103	37	
Age(years)			0.338
	69.0 ± 10.5	67.6 ± 9.6	
Pretreatment KPS			0.002
Median(range)	80 (50–100)	100 (50–100)	
Tumor origin			0.002
lung	121	68	(lung vs. non-lung)
breast	10	1	
colon	8	1	
stomach	4	0	
kidney	1	2	
esophagus	3	0	
other	7	0	
Mutation status in lung cancer			0.048
EGFR positive	28	21	
ALK positive	3	7	
EGFR/ALK negative	52	26	
NA	38	14	
Treatment			0.695
SRS	59	37	
SRS and SRT	42	13	
SRT	53	22	
Tumor number			0.248
median/average/range	2.16/1/1–9	2.08/1/1–9	
single	85	46	
multiple	69	26	
Tumor volume (cc)			0.881
median/average/range	0.3/2.13/0.01–26.52	0.31/1.76/0.02–33.51	
Control of primary tumor			0.001
yes	52	41	
no	102	31	
Extracranial metastasis			<0.001
yes	79	20	
no	75	52	
RPA class			<0.001
I	5	8	
II	105	55	
III	44	9	

EGFR: epidermal growth factor recptor, ALK: anaplastic lymphoma kinase, NA: not available.

**Table 3:** Logistic regression analysis for long-term survival.

Factor	Odds Ratio	95%CI	*p*-Value
Sex (female:vs. male)	1.94	1.050–3.60	0.0345
Age	1.02	0.989–1.05	0.226
Number of metastasis	1.1	0.575–2.09	0.782
Pretreatment KPS	2.54	1.150–5.59	0.021
Control of primary cancer	0.638	0.332–1.22	0.176
Extracranial metastasis	1.56	0.67–3.62	0.019
HR:hazard ratio			
CI:confidence interval			

**Table 4:** Summary of SRS/fSRT for long-term survivors after SRS and fSRT.

Author (Year)	Modality	Number of Pt	LTS	OS (Range)	Factors in LTS	Cause of Death	Complication
Kondziolka (2005) [6]	GK	44 /681 pts (6.5%)	≥4 years	68 months (48–156 months)	Higher pre-KPS Fewer brain metastases Less extracranial disease	1 pt: brain 27 pts: systemic cancer	4 pts (9%): permanent neurological deficits 6 pts (13.6%): salvage surgery
Yamamoto (2013) [9]	GK	167 pts (8.4%)	≥3 years	49.9 (36–142 months)	NA	16 pts: brain 76 pts: systemic disease	17 pts (10.1%): all complications 13 pts (7.8%): salvage surgery
Cohen-Inbar (2016) [10]	GK	92 pts	≥2 years	Survival rate 3 years: 73.7% 4 years: 51.8% 5 years: 41.7%	NA	NA	7 pts (7.6%): salvage surgery
Gogineni (2018) [8]	CK (86%) Others (14%)	132 pts (11.8%)	≥2 years	NA	NA	NA	NA
Siddiqui (2020) [7]	GK	198 pts	≥ 1 years	25.2 months (22.9–28.4 months) 2years: 52.8% 3 years: 33.4% 4 years: 22.1% 5 years: 16.6%	NA	NA	10 pts (5.1%): salvage surgery for radiation necrosis
This study (2022)	Linac based	72 pts (31.8%)	≥2 years	43 months (36–73) 3 years: 59.1% 4 years: 49.6% 5 years: 40.7%	Female High pre-KPS no extracranial metastasis	1 pt: brain 43 pts: systemic cancer	13 pts (18.0%): salvage surgery for 7 recurrences, 5 radiation necrosis, and 1 cyst formation

GK: Gamma Knife, pt: patient, mets: metastases, M:moth, CK: Cyber Knife, OS: overall survival, NA: not available. SRS/fSRT: stereotactic radiosurgery and fractionated stereotactic radiotherapy, LTC: local tumor control KPS: Karnofsky peformance score, BED: biologically effective dose.

## Data Availability

Data sharing is not applicable to this article as no datasets were generated or analyzed during the current study.
